# A new model of speciation

**DOI:** 10.1093/nsr/nwy111

**Published:** 2018-10-08

**Authors:** Loren H Rieseberg

**Affiliations:** 1 Department of Botany and Biodiversity Research Centre, University of British Columbia, Canada; 2 Reviewer of NSR

In the first paragraph of ‘On the origin of species’, Darwin [1] refers to speciation as that ‘mystery of mysteries’. Although the means by which new species arise is no longer mysterious, some puzzles remain. One such puzzle, which is addressed in a recent paper from Chung-I Wu's group on mangrove speciation [2], concerns the popular allopatric speciation model. In this model, which is widely viewed as the most common form of speciation, geographically isolated populations diverge to the point at which successful reproduction between them is no longer possible. However, there are too few geographical features that can offer stable isolation over long enough periods of time to account for the number of coexisting species that we see in the world today. Likewise, population genomic data reveal that a surprisingly high fraction of species that have been analyzed so far exhibit footprints of hybridization in their genomes. While models of speciation with gene flow (e.g. parapatric and sympatric speciation) can account for such a signature of hybridization, the widespread presence of postzygotic isolating barriers, even in taxa that currently hybridize, implies that periods of allopatry during divergence are common as well.

The mixing–isolation–mixing model (MIM) proposed by Ziwen *et al.* [2] offers a possible resolution to this conundrum. As suggested by the name, the model suggests that speciation often involves repeated cycles of admixture followed by geographical isolation, until full isolation is ultimately achieved. Such a model is difficult to prove based on population genomic data alone, so the authors have compiled a comprehensive array of genomic, geohistorical and theoretical data to support their proposed model in the mangrove system.

As with most concepts in evolutionary biology, the MIM model is not completely new. It bears some resemblance, for example, to Ehrendorfer's differentiation–hybridization cycles [3] and Rattenbury's cyclic hybridization model [4]. However, Ehrendorfer felt that hybridization would break down existing differentiation and that a new round of differentiation would then emerge from the hybrid complex. Thus, his views are arguably more similar to Seehauzen's proposal [5], that adaptive radiations might spring from the wide diversity generated by hybrid swarms, than to MIM. Rattenbury placed greater emphasis on the role of hybridization in re-acquiring key adaptations that would allow them to survive climate oscillations than on the importance of allopatric periods in generating postzygotic reproductive isolation. More generally, there has been widespread recognition since the 1970s that many taxa have experienced repeated episodes of allopatry and parapatry due to cyclic variation in climate. However, the emphasis of this work was more on phylogeography and hybrid zone dynamics than on a particular speciation mechanism such as MIM. It is also important to note that none of these earlier authors, to my knowledge, viewed hybridization–differentiation cycles as a solution to the conundrum of too little allopatry to account for patterns of species richness. Thus, I view the MIM proposal as a novel and important contribution to the field of speciation.
